# Assessing Caregiver Person‐Centeredness and Task‐Centeredness of Mealtime Care: Development and Psychometric Testing of Clinically Practical Scales

**DOI:** 10.1002/alz70858_104196

**Published:** 2025-12-26

**Authors:** Wen Liu, Yelena Perkhounkova, Maria Hein

**Affiliations:** ^1^ The University Of Iowa, Iowa City, IA, USA; ^2^ University of Iowa, Iowa City, IA, USA

## Abstract

**Background:**

Person‐centered mealtime care (PCMC) is defined as mealtime assistance prioritizing accommodations of individual abilities, needs, and preferences and engaging individuals in eating through dyadic interactions and environmental stimulations. Task‐centered mealtime care (TCMC) is defined as mealtime assistance prioritizing completion of eating activities without adequate considerations of individual needs and preferences. It is critical to maximize PCMC and minimize TCMC to optimize mealtime care quality and outcomes for people with dementia requiring mealtime assistance. Few tools are valid and clinically practical to measure PCMC and TCMC. This study adapted the Cue Utilization and Engagement in Dementia (CUED) video‐coding scheme, a novel and valid but resource‐intensive tool, into two observational scales to assess PCMC and TCMC, and examined their structural validity.

**Methods:**

*N* = 424 videos of dyadic mealtime interactions (mean duration = 25.8 minutes, range=1‐142 minutes) were coded using CUED. Coded behaviors were categorized based on their frequency: 0 (not observed), 1 (observed 1‐2 times), and 2 (observed ≥3 times). Several CUED items were combined based on content relevance, redundancy, and distributions, resulting in 16 PCMC items and 5 TCMC items. From the 424 videos, 20 random splits were created for exploratory (EFA) and confirmatory factor analyses (CFA) of the PCMC items. Internal consistency of the five TCMC items was examined with Cronbach's alpha

**Results:**

The videos captured *N* = 66 staff (age mean = 39.6 years, 84.9% female, 22.7% non‐white including 15.2% black, 16.9% Hispanic) in *N* = 10 nursing homes. For PCMC, 1‐ and 2‐factor structures with 8‐9 items were suggested by EFA. An 8‐item 1‐factor scale (Cronbach alpha=.75) was chosen based on conceptual interpretability, CFA model fit, internal consistency, and ease of use. For TCMC, a 3‐item scale (Cronbach alpha=.32) was identified.

**Conclusions:**

The 8‐item person‐centered mealtime care scale and 3‐item task‐centered mealtime care scale were developed. Future testing is needed using the scales for direct observations of mealtime care in nursing homes to establish validity and clinical utility.

